# The Neuroendocrine-Immune Regulation in Response to Environmental Stress in Marine Bivalves

**DOI:** 10.3389/fphys.2018.01456

**Published:** 2018-11-13

**Authors:** Zhaoqun Liu, Meijia Li, Qilin Yi, Lingling Wang, Linsheng Song

**Affiliations:** ^1^Liaoning Key Laboratory of Marine Animal Immunology, Dalian Ocean University, Dalian, China; ^2^Functional Laboratory for Marine Fisheries Science and Food Production Processes, Qingdao National Laboratory for Marine Science and Technology, Qingdao, China; ^3^Liaoning Key Laboratory of Marine Animal Immunology and Disease Control, Dalian Ocean University, Dalian, China

**Keywords:** marine bivalve, environmental stress, neuroendocrine system, immune response, physiological activities

## Abstract

Marine bivalves, which include many species worldwide, from intertidal zones to hydrothermal vents and cold seeps, are important components of the ecosystem and biodiversity. In their living habitats, marine bivalves need to cope with a series of harsh environmental stressors, including biotic threats (bacterium, virus, and protozoan) and abiotic threats (temperature, salinity, and pollutants). In order to adapt to these surroundings, marine bivalves have evolved sophisticated stress response mechanisms, in which neuroendocrine regulation plays an important role. The nervous system and hemocyte are pillars of the neuroendocrine system. Various neurotransmitters, hormones, neuropeptides, and cytokines have been also characterized as signal messengers or effectors to regulate humoral and cellular immunity, energy metabolism, shell formation, and larval development in response to a vast array of environmental stressors. In this review substantial consideration will be devoted to outline the vital components of the neuroendocrine system identified in bivalves, as well as its modulation repertoire in response to environmental stressors, thereby illustrating the dramatic adaptation mechanisms of molluscs.

## Introduction

Bivalvia are a class of molluscs, which have their soft bodies enclosed by a pair of calcified shells (Vokes, 1980; Morton, 2008). There are probably as many as 9,200 living species, which can be classified into 106 families and 1,260 genera. More than 80% of the species are found everywhere in the ocean, from intertidal zones to the deep blue sea, making them a vital pillar of the marine ecosystem (Hudson, 2002). Many bivalves are vital fishery and aquaculture species, and they are also ideal for studying neurobiology, immune mechanism, biomineralization, and adaptation to coastal environments under climate change (Lovatelli, 2006).

Over millions of years of evolution, marine bivalves have evolved sophisticated stress adaptation mechanisms to face harsh and dynamically changing environments. For example, exposure of the adult Sydney rock oyster *Saccostrea glomerata* to elevated pCO_2_ (the partial pressure of carbon dioxide) caused carry-over effects (Parker et al., 2012). The gene family of HSP70 (heat shock protein 70) and inhibitor of apoptosis (IAP) are significantly expanded in the Pacific oyster *Crassostrea gigas*, which is beneficial to the oysters’ successful adaptation to fast-changing environments in their living habitats (Zhang et al., 2012). Recently, stress response mechanisms modulated by the neuroendocrine system have attracted increasing attention toward marine bivalves. Molluscs are the most primitive creatures, which have developed a complete neuroendocrine-immune (NEI) system (Ottaviani and Franceschi, 1997). Various hormones, neurotransmitters, as well as their receptors and key enzymes, involved in their synthesis have been identified in marine bivalves (Song et al., 2015; Wang et al., 2018). Accumulating evidence reveals that the neuroendocrine system is indispensable in response to various environmental stressors by modulating immune activities, energy allocation, growth, and locomotion (Lacoste et al., 2001a,b,c). Marine bivalves are regarded as suitable models for investigating the fundamental mechanisms of neuroendocrine modulation in response to environmental stressors (Malagoli et al., 2017).

Recently, the diversity of marine bivalves is considered to be under severe threat owing due to worldwide climate change (Burge et al., 2014). Abiotic challenges such as temperature and ocean acidification, as well as biotic challenges such as bacteria and virus, all pose serious threats. Over the past century, ocean surface temperature has increased, and it is forecasted to continue increasing based on projections of several climate change models (Hansen et al., 2006; Lima and Wethey, 2012). Ocean acidification caused by excessive emission of carbon dioxide (CO_2_) has become a death threat to marine bivalves because of their limited ability to adjust the ionic balance of hemolymph (Thomsen et al., 2010; Melzner et al., 2011; Heinemann et al., 2012) and acute sensitivities (Barton et al., 2012; Hettinger et al., 2012, 2013; Waldbusser et al., 2013). Therefore, it is urgent and worthy to explore the stress response mechanisms of marine bivalves, which will contribute to the conservation of their biodiversity and prosperity. This review summarizes the research progress in the past 30 years involving major components of the neuroendocrine system identified in marine bivalves, its response features to environmental challenges, and regulation patterns on immunological and physiological activities, thereby contributing to a better understanding of the adaptation mechanisms of marine creatures and to a fast-changing ecosystem.

## Molecular Components of the Neuroendocrine System in Marine Bivalves

**FIGURE 1:**
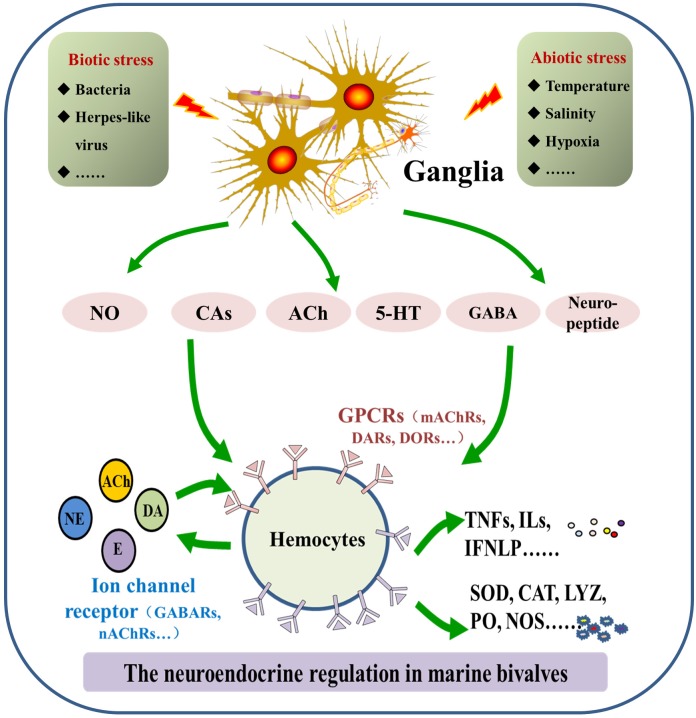
Molecular features of the neuroendocrine-immune (NEI) system in marine bivalves. The NEI system in marine bivalve mainly consists of neurotransmitters/hormones/neuropeptides, corresponding receptors and synthesizing/degrading enzymes.

Molecular components of the neuroendocrine system are highly conserved in marine bivalves and vertebrates, even though the functionally differentiated organs, such as the brain, thymus, and spleen, do not appear in bivalves. Thus far, catecholaminergic, cholinergic, enkephalinergic, serotoninergic, gamma-aminobutyric acid-ergic (GABAergic), and neuropeptide systems have been characterized for marine bivalves, which share a similar molecular basis as their counterparts in higher forms of life (Wang et al., 2018). However, there are still some unique features in marine bivalves’ neuroendocrine system. The binding specificity of receptors for neurotransmitters and hormones, most of which are G protein coupled receptors (GPCRs), is relatively low when compared with vertebrates (Reboul and Ewbank, 2016). In this section, molecular features of the neuroendocrine system identified in marine bivalves are described (Figure 1).

### The Catecholaminergic System

The catecholaminergic system contains major components of catecholamines (CA), CA metabolic enzymes, and CA receptors (Kvetnansky et al., 2009). The family of CA consists of dopamine (DA), norepinephrine (NE), and epinephrine (E), which was first discovered at the beginning of the 20th century (Sabban and Kvetnansky, 2001; Kvetnansky et al., 2009). Available evidence has demonstrated the presence and bioactivity of DA, NE, and E in many marine bivalves (Goh and Davey, 1976; O’Connor et al., 1982; Franchini et al., 1985; Pani and Croll, 1995). They are abundantly distributed in tissues including ganglion, hepatopancreas, hemocytes, and serum, with the highest levels detected in ganglion and hemocytes (Liu et al., 2018a). So far, four crucial CA metabolism enzymes, including phenylalanine hydroxylase (PAH) (Zhou et al., 2012a), L-DOPA decarboxylase (DDC) (Zhou et al., 2011d), dopamine beta hydroxylase (DBH) (Zhou et al., 2011c), and monoamine oxidase (MAO) (Zhou et al., 2011a), have been characterized in marine bivalves, performing catalyzing activities similar to their vertebrate counterparts. At the same time, adrenoceptors were also characterized in scallops *Chlamys farreri* (Zhou et al., 2013) and *Argopecten irradians* (Wang et al., 2009), as well as the oyster *C. gigas* (Lacoste et al., 2001a, 2002; Liu et al., 2018b). These findings indicate that marine bivalves occupy holonomic catecholaminergic neuroendocrine networks, which share structural and functional similarities with those in mammals. More importantly, it was reported that *Caenorhabditis elegans* only has a dopaminergic neuroendocrine system (Suo et al., 2004; Felton and Johnson, 2014). Thus, the catecholaminergic neuroendocrine system identified in molluscs should be considered the most primitive in structure but complicated in function during evolution, which makes molluscs the ideal model for the study of comparative neuroendocrinology.

### The Cholinergic System

Acetylcholine (ACh) is a neurotransmitter synthesized by choline acetyltransferase (ChAT) and hydrolyzed by acetylcholinesterase (AChE) (Deiana et al., 2011). Recently, the cholinergic nervous system was identified and characterized in marine bivalves (Shi et al., 2014). Enzyme activities of AChE were observed in hemocyte lysis of *C. gigas* and in tissues including gill, mantle, gut, and muscle of *C. hongkongensis* (Zha et al., 2013). One AChE was also identified in *C. farreri* (Shi et al., 2012). In addition, two nicotinic acetylcholine receptor alpha subunits (*Cf*nAChRα1 and *Cf*nAChRα2) were identified in *C. farreri*, and a homolog of muscarinic ACh receptor (*Cg*mAChR) was identified in *C. gigas* (Liu et al., 2016b). Furthermore, the cholinergic system is considered to be the most conserved neuroendocrine system among all phyla. Acetylcholine occurs in primitive invertebrates such as Sipunculida, Onychophora, and Tunicata (Florey, 1963) and can be detected in larval developmental stages as early as trochophore (Shi, 2012). The conservation in structure and function of the cholinergic neuroendocrine system indicates its central role in physiological and immunological regulation of marine bivalves.

### Nitric Oxide

Nitric oxide (NO) is a gaseous neurotransmitter synthesized by nitric oxide synthase (NOS) from L-arginine. It contains five cofactors including nicotine adenine dinucleotide phosphate (NADPH), flavin adenine dinucleotide (FAD), flavin mononucleotide (FMN), tetrahydrobiopterin (BH4), and heme (Knowles and Moncada, 1994). Nitric oxide and NOS have been detected in hemocytes of several bivalves including oyster, clam, and mussels (Torreilles and Romestand, 2001; Ivanina et al., 2010; Jiang et al., 2013a,b), suggesting that it may be crucial for immune regulation since hemocytes are the most important immunocytes in marine bivalves. Nitric oxide synthases in mammals can be classified into three major types – inducible NOS (iNOS), neuronal NOS (nNOS), and endothelial NOS (eNOS) – based on structural and functional features (Alderton et al., 2001). However, multi-isoform constitution of the NOS family is not found in marine bivalves. A unique NOS was identified in scallops and oysters, which was similar to nNOS in structure, to both nNOS and iNOS in biochemical characteristics, and to iNOS in immunological features (Jiang et al., 2013b, 2016). In arthropods, there is only one isoform of NOS (Jiang et al., 2013b), which implies that the differentiation of the NOS family might not have occurred before arthropods appeared. Moreover, only one mRNA transcript and several proteins forms of NOS were observed in bivalves. These results demonstrate the NOS family has not been well defferentiated in molluscs might be the milestone for the evolution of a neuroendocrine system.

### The Serotoninergic System

Serotonin, also known as 5-hydroxytryptamine (5-HT), is synthesized by tryptophan hydroxylase from tryptophan (Qi et al., 2016). Serotonin is one of the most well known neurotransmitters, first identified in clams in 1957 (Welsh, 1957). Contents of 5-HT in the nervous system were much higher in bivalves than in other invertebrate species (Bogdanski et al., 1956). Receptors of 5-HT have also been identified in bivalves *Mizuhopecten yessoensis* (Tanabe et al., 2010) and *C. gigas* (Jia et al., 2018). These receptors exhibited a high affinity to 5-HT and could relay signals via the mediation of cAMP (Jia et al., 2018). Serotonin is an ancient neurotransmitter has been identified as the early origin of the nervous system for both vertebrates and invertebrates (Berger et al., 2009). Molecular components of the serotoninergic system are highly conserved during evolution, while its development and biological functions vary significantly (Hay-Schmidt, 2000). Most studies related to the serotoninergic system are focused on its role in the larval development of marine bivalves, with an emphasis on the induction of metamorphosis.

### Neuropeptide

Neuropeptides refer to a vast array of short or long polypeptides, which are secreted by neurons, and work as regulatory molecules (Jekely, 2013). Neuropeptides are one of the most important components of the NEI system in marine molluscs and have been characterized in several molluscan species. For example, 74 putative neuropeptide genes were identified in Akoya pearl oysters, such as*Pinctata fucata*, and Pacific oysters, such as *C. gigas*, including three newly identified neuropeptide precursors PFGx8amide, RxIamide, and Wx3Yamide (Zhang et al., 2012; Stewart et al., 2014). Two GnRH-related peptides (CgGnRH-A and CgGnRH-G) were characterized by mass spectrometry from extracts of the visceral ganglia of *C. gigas* (Bigot et al., 2012). Both CgsNPFR (short neuropeptide F receptor)-like receptors and LFRFamide are highly expressed in the central nervous system of oysters (Bigot et al., 2014). Moreover, leucine (Leu) and methionine-enkephalin ([Met^5^]-ENK) were measured in hemocytes of *C. gigas* (Liu et al., 2008), and [Met^5^]-ENK occurred firstly on the marginal area of the dorsal half of D-hinged larvae during the developmental stage (Liu et al., 2015b). Receptors of ENK were also widely expressed in the important tissues of oysters (Guo et al., 2013; Liu et al., 2015a). These findings, including the comparative bioinformatical information, indicate that evolutionary origins of neuropeptide systems might have already emerged when protostomes and deuterostomes shared one common origin (Elphick et al., 2018), and multiple forms of neuropeptides and their receptors have evolved through the processes of genome duplication, gene duplication, and point mutation.

### The GABAergic System

The GABAergic system is predominantly an inhibitory system and contributes to homeostasis in the nervous system (Murphy et al., 2005; Shen et al., 2014). Apart from the nervous system, it also exists in immune cells such as monocytes and macrophages to exert a profound effect on immune function (Dionisio et al., 2011). The GABAergic system is composed of GABA synthase (glutamic acid decarboxylase, GAD), GABA catabolism enzyme (GABA transaminase, GABA-T), GABA transporters (GAT), and GABA receptors (Dionisio et al., 2011), and some of them have been discovered in marine bivalves. For example, one GAD homolog (CgGAD) was cloned from *C. gigas*, which was dominantly expressed in granulocytes to promote the production of GABA (Li et al., 2016b). Gamma-aminobutyric acid was also detected in the hemolymph of *C. gigas*, which performed inhibitory immunomodulation (Li et al., 2016a). Glutamic acid (Glu) is one of the most important neurotransmitters in ctenophore (Moroz et al., 2014) and is also characterized in *C. gigas*. It is implied that amino acid neurotransmitters might play a more important role in lower forms of life such as bivalves and ctenophore than in vertebrates. However, the GABAergic system in marine bivalves is still not well understood, and most research is focused on GABA receptors because they are a major site of action of the cyclodiene class of insecticides (Lunt, 1991).

## Response of the Neuroendocrine System to Environmental Stressors

The neuroendocrine system exhibits a quick response against external stimuli and modulates early stage stress response to maintain homeostasis in vertebrates (Shaliapina, 1996). As sessile organisms, marine bivalves are exposed to varying physical and physiological conditions. Both biotic threats from bacteria, viruses, and parasites, and abiotic threats such as extreme temperatures, osmotic changes, ocean acidification, and pollutant chemicals, are stressors for bivalves, and their neuroendocrine systems are also remarkably sensitive to these stressors. The neuroendocrine system can be triggered in response to thermal, osmotic, and bacterial stimuli, and then it conducts a series of modulation on immunological and physiological activities. In this section, response of the neuroendocrine system to environmental stressors will be discussed.

### Activation of the Neuroendocrine System in Response to Biotic Stressors

Marine bivalves are filter-feeding creatures that face tremendous exposure to microbial pathogens including bacteria, viruses, and protozoan parasites (Zhang et al., 2012) and have a notable immune defense mechanism, which can be modulated by the neuroendocrine system. Catecholaminergic, cholinergic, GABAergic, NO, and neuropeptide neuroendocrine systems in marine bivalves can be triggered immediately after pathogen invasion, activating the synthesis of various neurotransmitters. For example, mRNA expression levels and enzyme activities of ChAT and DBH in hemocytes enzymes for the synthesis of ACh and NE were significantly elevated at 1 h after lipopolysaccharide stimulation (LPS), while activities of AChE and MAO, two enzymes essential for the metabolic inactivation of ACh and NE, were inhibited (Liu et al., 2018a). With the acceleration of the synthesis process, a large amount of neurotransmitters are produced and released. It was reported that concentrations of NE, E, and DA increased significantly after the *scallop C.* farreri was challenged with the bacterium *V. anguillarum* (Zhou et al., 2011b). Gamma-aminobutyric acid was found to exist in the hemolymph of *C. gigas*, and its concentration decreased slightly from 8.00 ± 0.37 mmol L^-1^ at normal condition to 7.73 ± 0.15 mmol L^-1^ at 6 h after LPS stimulation (Li et al., 2016a). These newly synthesized neurotransmitters bind to their specific receptors to induce immunological and physiological modulation. Interestingly, the expression of these receptors is also activated under stress (Guo et al., 2013). These results reveal that the neuroendocrine system in marine bivalves is sensitive to biotic threats and can be quickly activated upon stress to release various neurotransmitters. Meanwhile, hemocytes are capable of adjusting the expression of neurotransmitter receptors, inducing an appropriate intensity of immune regulation. For example, LPS stimulation could induce the oyster *C. gigas* to release ENK to upregulate immune response in a very short period of time. However, at the later stage, NE instead of ENK was produced to downregulate immune response and restore it to normal level (Liu et al., 2017b). Such a neuroendocrine regulatory mechanism can effectively eliminate invading pathogens and simultaneously maintain inner homeostasis.

In addition, the production of cytokines can also be triggered in response to biotic stressors. It was reported that LPS treatment could induce mRNA expression of tumor necrosis factor (TNF) in oyster hemocytes (Sun et al., 2014). The mRNA expression of oyster interferon-like protein (IFNLP) was dramatically prompted after poly (I: C) stimulation (Zhang et al., 2015), and the expression of interleukin 17 (IL-17) was significantly triggered in oyster hemocytes under the stimulation of *Vibrio splendidus* (Xin et al., 2015).

### Activation of the Neuroendocrine System in Response to Abiotic Stressors

Most marine bivalves live on intertidal rocky shores, both one of the harshest environments and an ecosystem most vulnerable to climate change (Helmuth et al., 2006; Mieszkowska et al., 2006; Harley, 2011). In order to survive in these habitats, marine bivalves have to evolve efficient adaptation mechanisms to deal with physical threats, including extreme temperatures, osmotic changes, ocean acidification, and pollutants. Accumulating evidence has proved that the neuroendocrine system in marine bivalves can efficiently respond to abiotic stressors, in which catecholaminergic and serotoninergic regulation play central roles. Previous studies found that the concentration of 5-HT in hemolymph increased dramatically at 1 day post air exposure. After treatment with extra 5-HT, apoptosis rate of oyster hemocytes declined significantly, while the activity of superoxide dismutase (SOD) in hemolymph increased significantly. During air exposure, apoptosis rate of oyster hemocytes and the concentration of H_2_O_2_ in hemolymph decreased significantly after the stimulation of 5-HT, while SOD activity increased significantly. Furthermore, survival rate of oysters from the 4th to 6th day after injection of 5-HT, was higher than that of control and air exposure groups (Dong et al., 2017). Unlike the serotoninergic system, the catecholaminergic system could respond to various challenges such as high temperature, salinity alternation, and bacterial challenge. For example, contents of NE and E in scallop were obviously upregulated under salt stress (Chen et al., 2008). In the oyster *C. gigas*, increased DA and NE were measured in hemocytes post mechanical stress (Lacoste et al., 2001c,d), while CA concentrations in the brain of the clam *D. trunculus* decreased significantly after pollution stress (Bresler et al., 1999). Compared with biotic factors, exploration of neuroendocrine regulation in response to abiotic factors in marine bivalves remains relatively insufficient, and further detailed investigations are required to understand the adaptation mechanism of marine bivalves to climate change.

## Neuroendocrine Modulation of Immune Response

**FIGURE 2:**
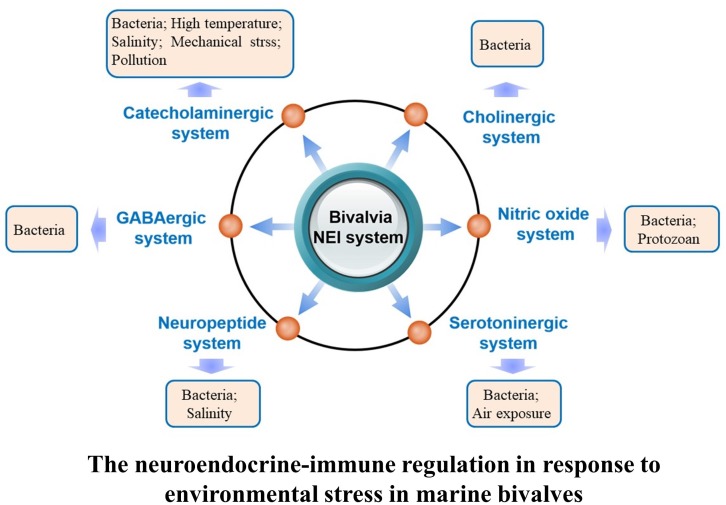
Regulation of the neuroendocrine-immune system in response to different kinds of environmental stressors in marine bivalve.

Once the neuroendocrine system of marine bivalves is activated in response to environmental stresses, molecules including neurotransmitters, hormones, and cytokines are released to regulate a series of physiological activities to meet the requirement for better adaptation and survival under various environmental conditions (Chen et al., 2008; Massarsky et al., 2011; Zhou et al., 2011b; Zhang et al., 2014). This section describes neuroendocrine modulation of immune response under environmental threats (Figure 2).

### Immunomodulation of the Developing Neuroendocrine System in Larvae

Neuroendocrine immunomodulation in marine bivalves appears as early as trochophore and D-hinged larvae during development. Transcriptomic analyses reveal that heat and bacterial stress can induce differential expression of genes related to monooxygenase activity, neuropeptide Y receptor activity, steroid hormone mediated signaling pathway, N-acetyltransferase activity, and neurotransmitter receptor activity (Liu et al., 2017a). In the early developmental period of oyster larvae, enkephalinergic system could modulate interleukin production, immune-related enzyme activities, and antibacterial responses (Liu et al., 2015b). Similarly, the catecholaminergic system in scallop larvae could also respond to immune challenges and conduct different patterns of immunomodulation in different developmental stages (Zhou et al., 2012b).

### Neuroendocrine Immunomodulation in Adults

Marine bivalves lack adaptive immunity and depend solely on innate immunity to fight against invading pathogens (Loker et al., 2004). The neuroendocrine system is one of the most intricate networks in the bivalves’ innate immune system, and has become the hotspot for the study of invertebrate immunity (Wang et al., 2018). Hemocyte is the most significant immunocyte in bivalves responsible for immune activities such as apoptosis, phagocytosis, cytokine production, and neurotransmitter release (Malagoli et al., 2017; Wang et al., 2017; Liu et al., 2018a). It is also the most important part of neuroendocrine immunomodulation (Liu et al., 2017b).

Recently, a simple but complete NEI network was revealed in the oyster *C. gigas*, which can modulate immune response via a “nervous-hemocyte”-mediated neuroendocrine immunomodulatory axis (NIA)-like pathway (Liu et al., 2016a, 2017b, 2018a). Such an NEI network can be activated in response to cytokines such as TNF or pathogenic stimulus such as LPS. The activated NEI system conducts immunomodulation by releasing neurotransmitters and hormones, which bind to their specific receptors and trigger intracellular second messengers including cAMP and Ca^2+^ (Zhou et al., 2013; Liu et al., 2015a). It is astonishing that different neurotransmitters in marine bivalves can impose diverse regulations. Acetylcholine and NE are able to downregulate immune response, while [Met^5^]-ENK upregulates immune response to regulate immune response by arising the expression of three TNFs (CGI_10005109, CGI_10005110, and CGI_10006440) and translocating two NF-κBs (*Cg*p65, CGI_10018142 and *Cg*Rel, CGI_10021567) between cytoplasm and nuclei of hemocytes (Liu et al., 2016a). Another exciting finding is that oyster hemocyte can *de novo* synthesize and release cholinergic and adrenergic neurotransmitters. Hemocyte-derived ACh/NE can execute a negative regulation of hemocyte phagocytosis with similar autocrine/paracrine signaling pathways identified in vertebrate macrophages (Liu et al., 2018a). These results suggest that bivalve hemocytes display similar immune and neuroendocrine functions as their vertebrate counterparts (e.g., macrophages) and play an indispensable role in autocrine/paracrine immunomodulation.

Apart from cellular immunity, the neuroendocrine system is also capable of regulating humoral immunity in adult marine bivalves by prompting the synthesis of immune-related enzymes and antibacterial peptides. In the scallop *C. farreri*, acute heat stress could induce the increase of SOD activity, and this trend could be reverted after blocking the binding activity of adrenergic receptors with the antagonist (Zhang et al., 2014). Apparently, both cellular and humoral immunities are elaborately modulated by the neuroendocrine system in marine bivalves, and such a highly efficient network plays a central role in stress response activities.

## Neuroendocrine Modulation of Physiological Activities

Except for immune response, some physiological activities in marine bivalves, such as growth, locomotion, reproduction, and biomineralization, are also under neuroendocrine control in response to environmental threats. It is reported that CA play an important role in the control of ciliary activity (Beiras and Widdows, 1995) and trigger settling and metamorphosis (Coon et al., 1985) or act as morphogens to participate directly in the early larval development of bivalves (Voronezhskaya et al., 1992, 1993; Buznikov et al., 1996). In this section, the discussion will concentrate on neuroendocrine modulation of physiological activities caused by environmental stressors.

### Modulation of the Neuroendocrine System on Redox Balance

The redox imbalance, usually caused by oxygen deficiency, is a common threat in near-shore regions. Glycogen and glucose metabolism are severely inhibited under redox imbalance (Gade, 1983). In order to deal with this, marine bivalves have evolved diverse and highly specialized strategies for surviving hypoxic episodes, including pathways that are efficient both in terms of ATP production and in minimizing H^+^ and toxic end product accumulation (Hochachka and Somero, 1984; Livingstone, 1991). It was reported that NO could relieve the redox status in oyster hemolymph by suppressing the level of reactive oxygen species (Jiang et al., 2013a). Moreover, after the injection of 5-HT during air exposure, the concentration of H_2_O_2_ in oyster hemolymph was significantly decreased, while SOD activity was significantly elevated (Dong et al., 2017). These results indicate that different kinds of neurotransmitters in marine bivalves are able to collaborate and conduct reciprocal modulation in response to external stimulus.

### Neuroendocrine Modulation on Shell Formation

Monoamines, a component of the neuroendocrine system, are critical for shell formation of marine bivalves. For example, a mussel-inspired route was reported to create carbonated bone hydroxyapatite from CaCO_3_ vaterite microspheres. When catechol-containing DA was incorporated during the mineralization of CaCO_3_, the oxidative polymerization of DA could stabilize the formation of spherical vaterite and transform it to carbonated hydroxyapatite crystals. In addition, DA was able to influence the level of conversion to carbonated hydroxyapatites (Kim and Park, 2010). Particularly, in the oyster *C. gigas*, DA together with its receptor CgD1DR-1 was involved in shell formation during larval development from trochophore to D-shape larvae, and CO_2_-induced ocean acidification could influence marine bivalves by inhibiting the DA–D1DR pathway to prohibit their shell formation (Liu et al., 2018b). Ocean acidification is a serious ecological problem and has become a huge threat to all marine calcifiers. Breakthroughs in the study of neuroendocrine modulation in response to ocean acidification in marine bivalves will help us to better understand and protect the marine ecosystem.

## Conclusion and Perspective

It has been reported that an increasing number of environmental stressors impact marine bivalves and cause severe mortalities in commercially or ecologically important species. Although some of these threats are imposed by the natural environment, more and more stressors can be linked to human activities. In order to adapt to the fast-changing environment, marine bivalves have evolved complex stress response strategies, in which neuroendocrine-immune regulation plays an indispensable role. Various neurotransmitters, hormones, neuropeptides, and cytokines work as signal messengers or effectors to regulate humoral and cellular immunity, energy metabolism, shell formation, and larval development under a vast array of environmental stressors, including pathogen infection, salinity alternation, high temperature, and ocean acidification. Hemocyte in marine bivalves is the most important component of such regulatory networks. It mediates the regulation of different effectors via specific receptors of neurotransmitters/hormones/neuropeptides/cytokines on the surface of the cell.

Despite recent advances, our understanding of the neuroendocrine system and its involvement in stress responses of marine bivalves is still very limited, and some gaps in techniques are restricting development in this field. For example, cell typing in marine molluscs is unclear, and little progress has been made in the study of neurobiology of marine molluscs. In future, urgent efforts to emphasize the key issues such as (1) molecular components and regulatory pathways of the neuroendocrine-immune system conserved between vertebrates and invertebrates; (2) variations of stress response modes of different bivalve species under different environmental challenges; and (3) molecular and cellular basis, as well as signal transduction pathways involved in neuroendocrine-immune regulation under various stressors, are needed. Furthermore, forecasting techniques and models based on neuroendocrine-immune regulation mechanism are also necessary for protection of the ecosystem and control of aquaculture diseases.

## Author Contributions

All the authors listed have made substantial, direct, and intellectual contributions to the work and approved it for publication.

## Conflict of Interest Statement

The authors declare that the research was conducted in the absence of any commercial or financial relationships that could be construed as a potential conflict of interest.
